# Visual timing abilities of a harbour seal (*Phoca vitulina*) and a South African fur seal (*Arctocephalus pusillus pusillus*) for sub- and supra-second time intervals

**DOI:** 10.1007/s10071-020-01390-3

**Published:** 2020-05-09

**Authors:** Tamara Heinrich, Andrea Ravignani, Frederike D. Hanke

**Affiliations:** 1 University of Rostock, Institute for Biosciences, Neuroethology, Albert-Einstein-Str. 3, 18059 Rostock, Germany; 2 Comparative Bioacoustics Group, Max Planck Institute for Psycholinguistics, 6525 XD Nijmegen, The Netherlands

**Keywords:** Timing, Interval timing, Pinnipeds, Sense of time, Time difference thresholds, Visual timing

## Abstract

Timing is an essential parameter influencing many behaviours. A previous study demonstrated a high sensitivity of a phocid, the harbour seal (*Phoca vitulina*), in discriminating time intervals. In the present study, we compared the harbour seal’s timing abilities with the timing abilities of an otariid, the South African fur seal (*Arctocephalus pusillus pusillus*). This comparison seemed essential as phocids and otariids differ in many respects and might, thus, also differ regarding their timing abilities. We determined time difference thresholds for sub- and suprasecond time intervals marked by a white circle on a black background displayed for a specific time interval on a monitor using a staircase method. Contrary to our expectation, the timing abilities of the fur seal and the harbour seal were comparable. Over a broad range of time intervals, 0.8–7 s in the fur seal and 0.8–30 s in the harbour seal, the difference thresholds followed Weber’s law. In this range, both animals could discriminate time intervals differing only by 12 % and 14 % on average. Timing might, thus be a fundamental cue for pinnipeds in general to be used in various contexts, thereby complementing information provided by classical sensory systems. Future studies will help to clarify if timing is indeed involved in foraging decisions or the estimation of travel speed or distance.

**Electronic supplementary material:**

The online version of this article (10.1007/s10071-020-01390-3) contains supplementary material, which is available to authorized users.

## Introduction

The sense of time is a fascinating sense as it is involved in numerous behaviours ranging from vocalisations to foraging as well as reproduction. Timing and its mental representation is linked to many perceptual and cognitive processes such as attention or memory (Matthews and Meck 2016) and, thus, is an essential and transversal aspect of animal cognition. In contrast to the classical senses, there is no specific organ responsible for receiving temporal information. Moreover, depending on the time scale or timing task, different brain areas are involved in the processing of temporal stimuli (for example in Buhusi and Meck 2005; Coull et al. 2011; Drayton and Furman 2018; Merchant and de Lafuente 2014). So far, the timing abilities of many animals including pigeons (Santi et al. 1998, 2007; Stubbs 1968; Yamashita 1986), rats (Church et al. 1976; Crystal 2015; Whitaker et al. 2003) or cats (Rosenkilde and Divac 1976) were investigated using different approaches, and interval timing, meaning the perception and processing of individual temporal intervals from milliseconds to several seconds, has caught most attention (Buhusi and Meck 2005; Droit-Volet et al. 2007; Matell and Meck 2000; Oprisan and Buhusi 2014; Richelle and Lejeune 1984).

Recently, we hypothesised that timing might be a parameter equally important to terrestrial and aquatic animals (Heinrich et al. 2016). In the aquatic environment, the information provided by the classical sensory systems might not be reliable under certain circumstances, rendering intrinsic parameters such as time even more valuable, for example, in the context of orientation and navigation. Regarding these aspects, time could help to determine distance travelled by keeping track of swimming velocity or it might be used to determine travel direction by interpreting the sun’s position. Well-developed timing abilities might also support the estimation of the energy gain per time during a foraging trip or the assessment of travel duration between food patches in aquatic animals in general and in marine mammals in particular. These parameters are crucial to be considered with respect to the optimal dive theory (for example Boyd and Croxall 1996; Cornick and Horning 2003; Foo et al. 2016; Heaslip et al. 2014), an extension of the optimal foraging theory (Krebs and Davies 1981; Doniol-Valcroze et al. 2011).

Whereas interval timing studies involving terrestrial animals are numerous (Buhusi and Meck 2005; Church 1984; Lejeune and Wearden 1991; Lejeune and Wearden 2006; Penney et al. 2008; Wearden 1991), only two studies addressed timing or timing-related aspects in marine mammals. First indirect evidence for timing abilities in pinnipeds was gathered in a rhythm experiment with a representative of the otariids, a California sea lion (*Zalophus californianus*; Cook et al. 2013; Rouse et al. 2015, 2016). The California sea lion showed the ability to entrain to an external beat, which, of course, requires a sense of time. The second study directly investigated the timing abilities of a phocid, a harbour seal (Heinrich et al. 2016). After a short learning process, the harbour seal successfully discriminated visually presented time intervals, and low time difference thresholds for time intervals ranging from 3 s to 30 s were found.

The present study is a direct continuation and extension of this first timing study involving harbour seals *(Phoca vitulina)*. First, we set out to directly compare the harbour seal’s timing abilities with the timing abilities of an otariid species, the South African fur seal *(Arctocephalus pusillus pusillus)*. This comparison seemed essential as phocids and otariids differ tremendously in their anatomy, amount of social interaction, lactation period, duration of pup care, foraging strategies, or different degrees of adaptation to the aquatic medium (Mellish et al. 1999; Schulz and Bowen 2004, 2005; Stephens et al. 2014); even within the phocid or otariid families, pronounced species-specific differences are discernible. The otariids could have evolved different or even better (visual) timing abilities as, for example, they might need timing in the short-range social interactions occurring more frequently and throughout the year in these social animals in comparison to the less social phocids. Alternatively, the timing performance of phocid and otariid species might not differ substantially as phylogenetically the pinnipeds have evolved separately only since 15 mio years (Berta 2018) in contrast to macaques and humans that diverged approximately 25 mio years ago but still show comparable timing performance (Mendez et al. 2011). For this reason, we tested a representative of the otariids, a South African fur seal, for its ability and sensitivity to discriminate time intervals from 0.2 s to 12 s.

Second, we extended the data set of the harbour seal (Heinrich et al. 2016) by assessing difference thresholds for millisecond time intervals, which is of interest in many respects. First we determined difference thresholds for time intervals shorter than 3 s, which was the shortest time interval tested in Heinrich et al. (2016). Thereby we intended to further characterise the sense of time in our model species. We tested two alternative hypotheses, namely whether in harbour seals (1) the difference thresholds follow Weber`s law even when short time intervals are included in the analysis or instead whether (2) sensitivity changes occur for short time intervals. The first hypothesis would predict a linear relationship between the difference thresholds and the time intervals; whereas, the latter hypothesis would most likely result in the timing performance being worse for very short time intervals resulting in a more U-shaped relationship between time interval and difference threshold. Timing studies involving other organisms make both predictions, in principle, plausible (for example Bangert et al. 2011; Bizo et al. 2006; Merchant and de Lafuente 2014). A change in sensitivity over a broad range of stimulus intensities also characterises other sensory abilities (Grondin 2012). Differences in performance for second versus millisecond time intervals might occur as the available experimental evidence suggests that sub- and supra-second time intervals are processed differently. On the one hand, different timing mechanisms including specific brain areas are involved in the perception and processing of temporal events of different time scales (Buhusi and Meck 2005; Merchant and de Lafuente 2014), and the firing latency (Mendoza et al. 2018) and rate (Merchant et al. 2011; Mita et al. 2009; Wang et al. 2018) of neurons in some brain areas vary depending on the duration of the temporal stimulus. On the other hand, it is assumed that time in the sub-second range is perceived immediately; whereas, the perception of longer time intervals includes conscious and cognitive processes (Lewis and Miall 2003; Merchant and de Lafuente 2014; Rammsayer 1999).

Millisecond timing abilities are also of interest regarding some seal behaviours such as avoiding collisions, which could be based on optic flow information (Gläser et al. 2014), or following prey at close distance, which might show fast evasive manoeuvres such as C-starts (for review Domenici and Hale 2019).

## Materials and methods

### Experimental animals

The experimental animals were a male harbour seal called `Luca` (13 years old at the beginning of experiments) and a male South African fur seal called `Fin` (5 years old at the beginning of experiments). Both animals were kept at the “Marine Science Center” of the University of Rostock, Germany, where they received approximately 50 % of their daily diet during the experimental sessions once or twice per day, five–seven days a week; the rest of the food was provided in training besides the experiments. Both seals were experienced in conducting visual experiments. However the harbour seal had already participated in numerous visual and visual cognitive experiments including the first timing experiment (Heinrich et al. 2016; Scholtyssek et al. 2008; Scholtyssek et al. 2013); whereas, the fur seal’s previous experience was limited to a brightness discrimination study (Scholtyssek and Dehnhardt 2013).

### Apparatus

**Fig. 1.**
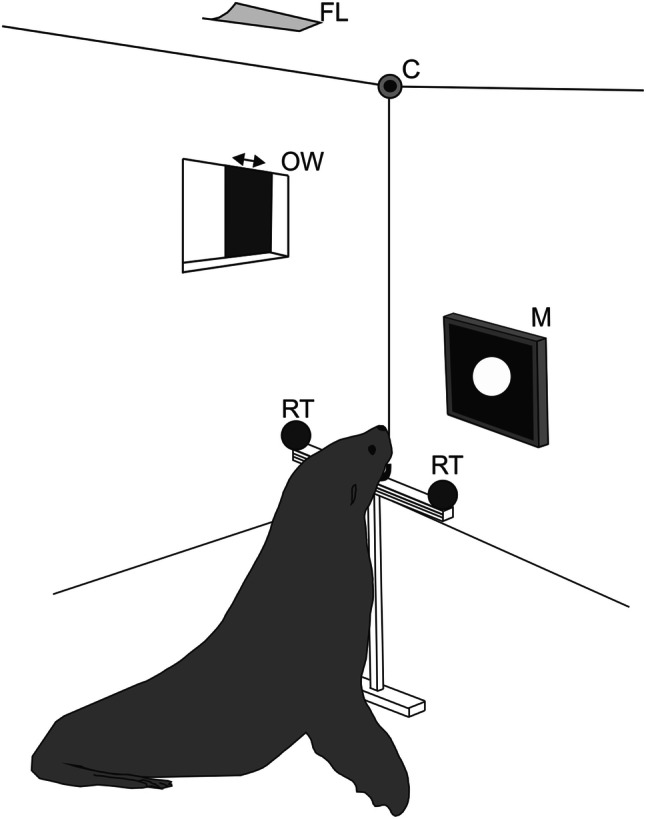
Experimental setup to investigate the timing abilities of a South African fur seal. The animal was stationing in a chin station at 50-cm distance from the monitor (*M*). On the monitor, the stimulus, a white circle on black background, was presented for a pre-programmed time interval. The animal indicated its answer by moving its head to one out of two response targets (*RT*). The response to the left target was correct after the presentation of the standard time interval (*STI*) and the response to the right target of the station was correct after the presentation of a longer comparison time interval (*CTI*). The experimenter was hiding in an observation room adjacent to the experimental chamber. During the session, the animal could then be observed via a camera (*C*) and was rewarded for every correct answer by opening the opaque slider of the observation window (*OW*) and providing a fish reward to the seal. A fluorescent lamp (*FL*) installed on the ceiling guaranteed constant illumination within the chamber

All experiments were conducted in an experimental chamber (3 m deep, 2 m wide, and 2 m high) to achieve a constant illumination via a fluorescent lamp (Standard FSL T8 36W 765 Radium, Wipperfürth, Germany). The illumination, measured with a luxmeter (Voltcraft VC 4 in 1, Multifunctional Environment Measuring Instrument, Conrad electronics AG, Wollerau, Switzerland), was 40 lx in the surrounding of the station of the harbour seal (a drawing of the harbour seal's station can be found in Heinrich et al. 2016). With the fur seal, we first worked with open chamber due to issues related to the motivation of this less experimentally experienced animal; thus, the illumination first was 240 lx at the station of the fur seal. Afterwards, we could continue with closed chamber which reduced the illumination to 60 lx (Fig. 1). The difference in illumination at the stations even in the closed chamber resulted from the different height of the animals’ experimental stations which was varied to enable both animals a natural body position during the experiment. For the harbour seal, the station consisted of a metal hoop affixed to a steel plate 6 cm above the bottom. For the fur seal, the station consisted of a jaw station attached to aluminium profiles (60 cm long, 5 cm wide) at a height of 68 cm (Fig. 1). The experimental stations ensured a constant distance of 50 cm to, and a constant viewing angle of the seals on the LCD monitor (Eizo, Flex scan S1721, 17”, refresh rate 60 Hz, Eizo Nanao Corporation, Hakusan, Ishikawa, Japan), on which the stimuli were presented (see “Stimuli”). Two response targets were fixed on both sides of the respective station devices. The animals moved their heads to one of these response targets after stimulus presentation (see “Procedure”).

During the experiment, the experimenter was hiding in an observation room adjacent to the experimental chamber. Both chambers, the experimental chamber and the observation room, were linked by a window. During each trial, the window was closed by a black opaque slider to prevent secondary cueing. It was opened for rewarding the animal after a correct answer. A camera (HD Logitech Webcam C270, Logitech GmbH, Munich, Germany), installed in the experimental room, allowed to observe the response behaviour of the animals during the session. All technical equipment was located in the observation room allowing the experiment to be operated from this room.

### Stimuli

The stimuli, displayed on the LCD monitor, were white-filled circles, 10.5 cm in diameter, on black background. The circle was presented either for the duration of a standard time interval (STI) or for a longer comparison time interval (CTI). The fur seal was tested with eight STIs: 0.2 s, 0.4 s, 0.8 s, 1.6 s, 3 s, 5 s, 7 s, and 12 s. The harbour seal was tested with five STIs: 0.1 s, 0.2 s, 0.4 s, 0.8 s, and 1.6 s; his thresholds for STIs ranging from 3 s to 30 s were already published (Heinrich et al. 2016). This list of STIs illustrates that, in the fur seal, difference thresholds could unfortunately neither be assessed for a 0.1 s STI nor for STIs longer than 12 s as the animal refused to work under these conditions; this generally very unsteadily cooperating animal was unmotivated to keep attention to the very short STIs and to wait for the end of stimulus presentation for long STIs.

The stimuli ranging from 0.2 s to 1.6 s were programmed and presented with PsychoPy 1.82.01 (Peirce et al. 2019; Peirce 2007; Peirce 2009) and the stimuli ranging from 3 s to 12 s were programmed and presented with Matlab R2012b (The Mathworks, Natick, Massachusetts, USA) and the Psychophysics toolbox 3.0 (Brainard 1997; Kleiner et al. 2007; Pelli 1997). We used Matlab to assess difference thresholds in the fur seal for STIs 3 s and longer allowing direct comparison with the harbour seal data from Heinrich et al. (2016). Psychopy was used for STIs 1.6 s and shorter as this programme was more accurate for these time intervals. The accuracy of stimulus presentation was measured with a CMOS camera (Phantom V12, Vision Research Wayne, NJ, USA) recording at 1,000 frames per second. Two human observers counted the number of frames between the frame on which the stimulus had just appeared and the first frame on which it had just disappeared. From the number of frames, the duration of the respective time interval could be calculated and compared to the programmed value. The duration of the time intervals programmed in Matlab deviated by on average 40.0 ± 8.9 ms from the programmed value, and the duration of the time intervals programmed in Psychopy deviated by on average 25 ± 4.7 ms from the programmed value.

### Procedure

Each trial started with the experimental animal stationing in its designated station in the experimental chamber. After guiding the animal to its station, the experimenter left the experimental chamber and entered the observation room. The opaque slider was closed after the experimenter had rechecked that the animal stationed correctly and paid attention to the monitor. Then stimulus presentation could be started. In each trial, only one type of stimulus was presented, either the STI or CTI. After the presentation of the respective stimulus for the pre-programmed time, the animal had to indicate its response by touching one of the two response targets. A correct answer was defined as the seal touching the left response target after the STI and the right response target after the CTI. If the response was correct, the slider was opened, and the animal was rewarded with fish. A wrong response was signalled by the experimenter with the German word for no “nein”, the slider stayed closed, and the animal had to station again. The next trial started after approx. 5 s. Only rarely the intertrial interval was prolonged by 2–3 s, for example, if the animal needed to be signalled to pay attention to the monitor or if external noise disturbed the experiment.

One session consisted of 30 trials, 15 of which featured the STI and the other 15 featured the CTI. The sequence with which the two types of stimuli were presented followed a pseudo-randomised scheme (Gellermann 1933).

The time difference threshold of the seals was determined using a modified staircase method. A detailed scheme illustrating the process of threshold determination can be found in Heinrich et al. (2016). In brief, the STI remained constant during the determination of one threshold, and only the CTI was decreased, if the animal had reached the preset learning criterion. The learning criterion was defined as a minimum number of 23 correct trials in a session of 30 trials in total (76.7%, Chi square test: *p* < 0.01). This performance had to be achieved in two consecutive sessions to meet the learning criterion. If the animal reached the criterion, the duration of the CTI was decreased by either 1 s or 2 s or by halving the time difference between STI and CTI depending on the experimental situation (for details please see Supplementary material). If instead the seal did not reach the learning criterion, up to five sessions were conducted altogether before the threshold determination was ended. Five sessions were conducted as the training had previously revealed that the seal’s performance would not improve even if training was continued for ten sessions. However, if the seal achieved a performance of ≥ 76.7% correct choices in the fifth session, a sixth session was conducted. During this session, the seal could either meet the learning criterion, and threshold determination was continued by decreasing the CTI once more, or its performance again dropped below 76.7 % correct choices, and threshold determination for the respective STI was ended. Only after the determination of a difference threshold for one STI, a new STI was introduced and paired with suitable CTIs for the determination of the difference threshold for the new STI (for details about the sequence with which difference thresholds were determined for specific STIs, please see Supplement).

### Analysis

The difference threshold was defined as the time difference between STI and CTI that the animal was able to discriminate with a performance at 75 % correct choices. The difference threshold was calculated via linear interpolation from the mean performance of the last two consecutive sessions above 75 % correct choices and the mean performance of the first five consecutive sessions below 75 % correct choices. Additionally, we calculated the Weber fraction c as

c = $$\frac{\Delta S}{S}$$,

with ΔS being the difference threshold for a respective STI, and S, the corresponding STI. The Weber fraction c should be constant for the tested STIs, if Weber’s law is valid for time perception, either for part of or the full range of STIs.

Statistical analysis was performed in IBM SPSS Statistics Version 25 (IBM, Armonk, NY, USA).

## Results

### Performance of the South African fur seal

**Fig. 2:**
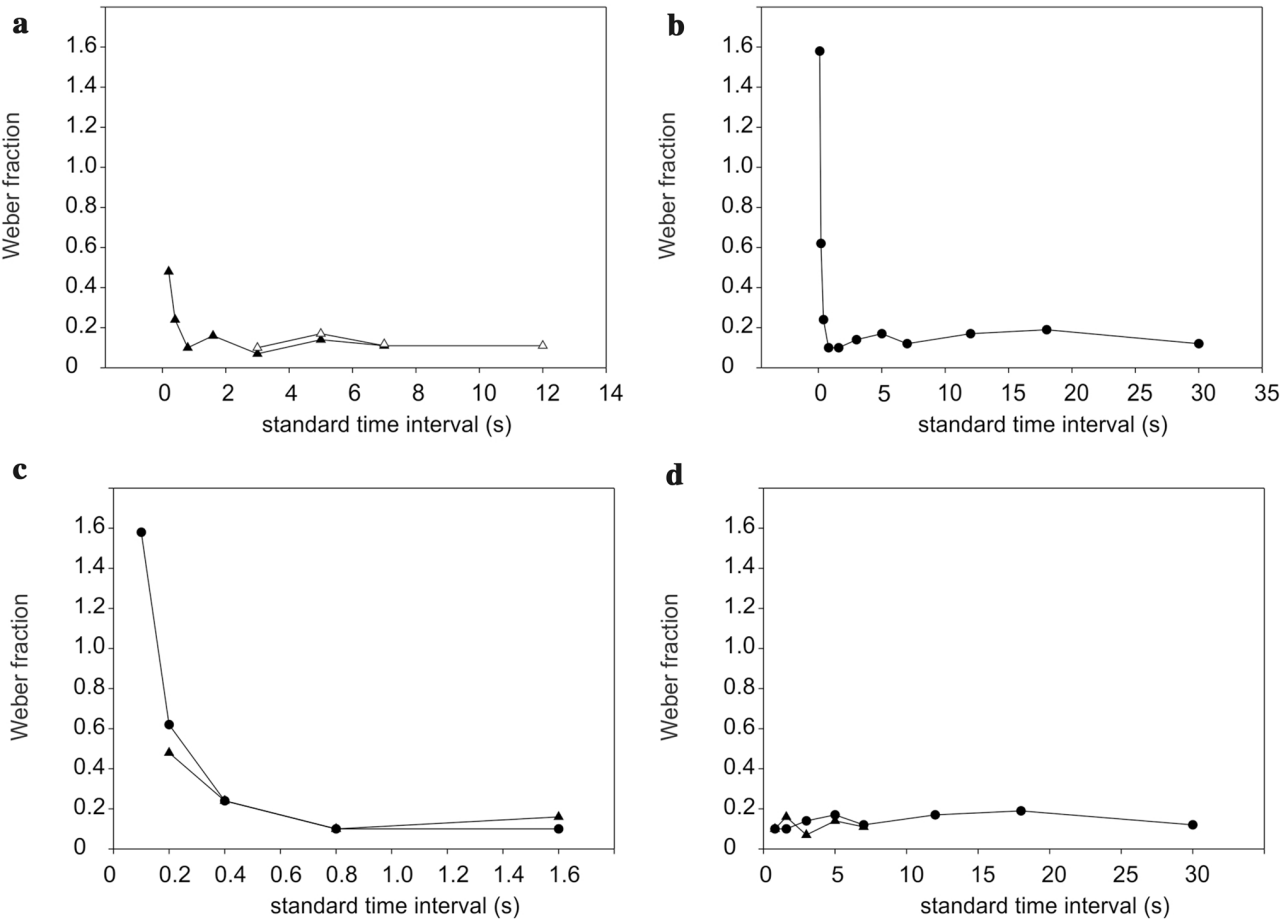
**a** Weber fractions of the South African fur seal for the tested standard time intervals (STIs) from 0.2 s to 7 s in closed chamber/low ambient light (filled triangles) and from 3 s to 12 s in open chamber/high ambient light (open triangles). **b** Weber fractions of the harbour seal for the STIs from 0.1 s to 30 s. The timing data for STIs 3 s to 30 s were adopted from Heinrich et al. (2016). **c** Weber fractions of the harbour seal for the tested STIs from 0.1 s to 1.6 s and of the South African fur seal for STI from 0.2 s to 1.6 s (closed chamber/low ambient light). **d** Weber fractions for the harbour seal and the South African fur seal in the time range in which a constant relationship between Weber fraction and standard time interval can be found, i.e. in which Weber’s law holds. Please note that for this comparison, the fur seal data obtained in closed chamber /low ambient light were taken which does not include a difference threshold for a STI of 12 s. Difference thresholds for STIs of 18 s and 30 s were only determined for the harbour seal. The data of the harbour seal are generally depicted with filled circles, the data of the fur seal with triangles

With the experimental chamber open, the South African fur seal was first asked to discriminate an STI of 5 s and a CTI of 15 s (details about the sequence of testing, the STIs and CTIs as well as the performance of the fur seal can be found in Supplement Tables 1 and 2). As the fur seal did not learn the experimental procedure with this stimulus combination, the CTI was reduced to 11 s. However, after a total number of 30 sessions with 856 trials with these two CTIs, including trials during which the fur seal was assisted in learning with the experimenter present and pointing at the correct response target, no learning effect was discernible. Consequently the STI was changed to 3 s and was tested against a CTI of 11 s. With this STI/CTI combination, the animal learned the task and reached the learning criterion in 207 trials. After one session of overtraining, the animal was even able to reach the learning criterion in the number of trials minimally required to meet the learning criterion with the new CTI of 9 s. The fur seal continued to respond with this high performance. Thus, we were able to collect a data set for the STIs of 3 s, 5 s, 7 s, and 12 s with open chamber resulting in difference thresholds of 0.32 s, 0.84 s, 0.77 s, and 1.3 s (Fig. 2a; Table 1). The corresponding Weber fractions for these STIs are 0.10, 0.17, 0.11 and 0.11 resulting in a mean Weber fraction of 0.12.

**Table 1:** Overview of the standard time intervals (STI, in s) and the difference thresholds (∆S, in s) as well as Weber fractions in the timing experiment with a harbour seal and a South African fur seal for different experimental conditions (closed versus open chamber)

	Harbour seal		South African fur seal			
	Closed chamber (40lx)		Closed chamber (60lx)		Open chamber (240lx)	
STI (s)	ΔS (s)	Weber fraction	ΔS (s)	Weber fraction	ΔS (s)	Weber fraction
0.1	0.16	1.58				
0.2	0.12	0.62	0.10	0.48		
0.4	0.09	0.24	0.10	0.24		
0.8	0.07	0.10	0.08	0.10		
1.6	0.16	0.10	0.25	0.16		
3	*0.42*^*a*^	*0.14*	0.21	0.07	0.32	0.10
5	*0.85*	*0.17*	0.71	0.14	0.84	0.17
7	*0.84*	*0.12*	0.78	0.11	0.77	0.11
12	*2.05*	*0.17*			1.30	0.11
18	*3.46*	*0.19*				
30	*3.70*	*0.12*				

^a^ The data set for the difference thresholds for the time intervals between 3 s and 30 s for the harbour seal was taken from Heinrich et al. (2016)

After intensive training in the closed chamber, we could work with the fur seal in the closed chamber. First, we replicated the difference thresholds for the STIs of 3 s, 5 s, and 7 s (Fig. 2a, Table 1). The resulting difference thresholds were 0.21 s, 0.71 s, and 0.78 s. The corresponding Weber fractions for these STIs were 0.07, 0.14 and 0.11 and resulted in a mean Weber fraction of 0.11. Thus, we did not find a significant difference between lighting conditions (one-sided unpaired t-test for independent samples *F*=0.020, *p*=0.2575). A difference threshold for the 12 s STI was not determined under these experimental conditions due to lack of motivation of the animal to work with long stimuli.

Subsequently, difference thresholds for the STIs 0.2 s, 0.4 s, 0.8 s, and 1.6 s were determined in the closed chamber. For these STIs, the difference thresholds decreased with increasing STI from 0.10 s for a 0.2 s STI to 0.10 s for a 0.4 s STI, and 0.08 s for a 0.8 s STI (Fig. 2c; Table 1). For the 1.6 s STI, the difference threshold was assessed as 0.25 s. The corresponding Weber fractions for the STIs 0.2 s, 0.4 s, 0.8 s, and 1.6 s were 0.48, 0.24, 0.1, and 0.16 resulting in a mean Weber fraction of 0.25.

In general, changes in STI had no influence on the fur seal’s performance; the fur seal continued to respond with high precision, and it often met the learning criterion after the minimum number of trials required to reach the learning criterion. Only for the STIs of 7 s (closed chamber), and 0.4 s (closed chamber), the animal needed four sessions to reach the learning criterion during acquisition.

The fur seal`s timing abilities showed a linear relationship between the STIs of 0.8 s and 7 s (Fig. 2d), which were tested under the same experimental conditions, meaning under low ambient luminance in the closed chamber, and the difference thresholds (*r*^2^ = 0.90). This implies that Weber`s law holds; the mean Weber fraction for this temporal range was calculated as 0.12.

### Performance of the harbour seal

The harbour seal transferred the experimental paradigm from long time intervals (Heinrich et al. 2016) to the first STI of 1.6 s tested in this study within five sessions (details about the sequence of testing, the STIs and CTIs as well as the performance of the harbour seal can be found in Supplement Table 3). Thereafter, when the sub-second STIs of 0.8 s and 0.4 s were introduced, it took the seal only the two sessions minimally required to reach the learning criterion during the acquisition of the initial discrimination of the specific STI. When introducing the STIs of 0.2 s and 0.1 s, the animal met the learning criterion in four and three sessions, respectively.

The difference thresholds decreased with increasing STI from 0.16 s determined for the 0.1 s STI to 0.12 s for the 0.2 s STI, 0.09 s for the 0.4 s STI and 0.07 s for the 0.8 s STI (Fig. 2b,c; Table 1). For a STI of 1.6 s, the difference threshold increased to 0.16 s. Consequently, the Weber fractions decreased from 1.58 for the 0.1 s STI to 0.62 for the 0.2 s STI, 0.24 for the 0.4 s STI, 0.10 for the 0.8 s STI, and 0.10 for the 1.6 s STI. The mean Weber fraction for this range of time intervals was calculated as 0.53.

The harbour seal`s timing abilities over the whole tested time range including the data set of Heinrich et al. (2016) showed a linear relationship between the STIs of 0.8 s and 30 s and the difference thresholds (*r*^2^ = 0.92; Fig. 2d). This implies that Weber`s law holds; for this temporal range, a mean Weber fraction of 0.14 was determined.

## Discussion

Our results demonstrate a high sensitivity of a harbour seal and a South African fur seal for the discrimination of time intervals over a wide temporal range.

Both seal individuals found access to the timing task relatively easily although, initially, the fur seal was not able to learn the timing task with a 5 s STI and a 15 s CTI or an 11 s CTI within 856 trials. However, after changing to a 3 s STI and an 11 s CTI, the fur seal only needed 207 trials to meet the learning criterion. For comparison, the harbour seal finished the acquisition phase after 232 trials in our first timing study (Heinrich et al. 2016). The difference in the rate of acquisition might be related to the fact that the harbour seal had already participated in numerous studies including visual as well as cognitive experiments (Scholtyssek et al. 2008; Scholtyssek et al. 2013). In contrast, the fur seal had only taken part in a brightness discrimination experiment (Scholtyssek and Dehnhardt 2013). Taking this limited experimental experience of the fur seal into account, we conclude that both individuals learnt the task relatively quickly which is supporting the assumption that time is a cue which pinnipeds are able to isolate fairly easily.

In general, Weber’s law holds for a wide range of time intervals in pinniped timing. The harbour seal’s timing performance can be characterised by Weber’s law from the 0.8 s STI to the 30 s STI with a mean Weber fraction of 0.14. In comparison, the fur seal's timing performance shows a constant Weber fraction from the 0.8 s STI to the 7 s STI with a mean Weber fraction of 0.12. It needs to be noted that for this analysis, only the fur seal’s thresholds obtained in the closed chamber were considered as these allow best comparison to the data set obtained with the harbour seal. For both animals, the Weber fraction for STIs below 0.8 s was not constant anymore; instead, the Weber fraction increased, the shorter the STI. A deviation of the linearity for low, and for high, intensities has already been shown for many sensory modalities (see for example Gescheider 1976). According to Gescheider (1976), the increase of the Weber fractions at low intensities might be explained by the difference thresholds for these intensities close to the threshold of perception being influenced by spontaneous neuronal activity, i.e. sensory noise. Thus, our data support hypothesis 2 (see “Introduction”) suggesting the timing performance to decrease with very short STIs.

Comparisons of the mean Weber fraction of 0.12 for the fur seal and 0.14 for the harbour seal, assessed for STIs from 0.8 s to 7 s (fur seal) or to 30 s (harbour seal) to the timing abilities of other species (Gibbon et al. 1997; Lejeune and Wearden 1991) demonstrates that we could document a high timing accuracy for the representatives of both pinniped species. The mean Weber fractions describing the timing abilities in pinnipeds suggest a comparable or even higher precision in comparison to the seal’s performances with stimuli of other sensory modalities (Dehnhardt and Kaminski 1995; Dehnhardt and Mauck 2008; Scholtyssek et al. 2008; Wieskotten et al. 2011).

This study also revealed that the timing ability of both pinnipeds is similar for the tested temporal range when the seals are trained in the closed experimental chamber. The fur seal’s timing performance can be characterised by a mean Weber fraction of 0.12 for STIs ranging from 0.8 s to 7 s in the experimental condition with closed chamber. Calculating the mean Weber fractions for the harbour seal for the STI ranging from 0.8 s to 7 s as tested for the fur seal and thereby combining the results of this and the first harbour seal timing study (Heinrich et al. 2016), a mean Weber fraction of 0.13 results. The subtle, but not significant difference (one-sided unpaired t-test for independent samples *F*=0.034; *p*=0.3265), between the mean Weber fractions of both pinniped species might be explained by small differences in the experimental setups, the time of the day when testing took place, motivation, age, or sexual maturity; these factors are known to influence human timing (Droit-Volet and Clement 2001; Droitt-Volet et al. 2007; Huang et al. 2012; Katsuura et al. 2007; Kuriyama et al. 2005; Morita et al. 2007). Motivation or cooperativeness definitely varied over the course of this study in the fur seal. However, it made threshold determination for some STIs impossible in this individual; whereas, the thresholds obtained did not differ significantly from the harbour seal’s threshold performance. It needs to be noted that, although previous studies documented an influence of ambient illumation on timing abilities (Delay and Richardson 1981; Huang et al. 2012; Katsuura et al. 2007), we found no significant differences between the calculated mean Weber fractions of the fur seal under different illumination. The absence of a significant influence of the different light conditions on timing sensitivity during the experiment could be an adaption to changing environmental conditions, with which pinnipeds have to cope with in their natural environment when, for example, leaving the bright surface and diving down to deeper and dark waters. In general, against one of our hypotheses, the different degree of adaptation of the two species to the marine environment or the general differences between the pinniped families does not seem to result in substantially different timing abilities.

It needs to be mentioned that we tested only one harbour seal and one fur seal individual. Thus, we cannot assess if their timing data are representative for the species as individual differences can occur within species. Future experiments could test more individuals of the species involved in this study, thereby increasing sample size, as well as of other pinniped species for further comparative insight into the timing abilities of pinnipeds.

Taken together, we have gathered evidence that representatives of two pinniped species possess a sense of time with which they can discriminate millisecond to second time intervals with high precision. The timing performance of the two seals stood out by (1) the acquisition being relatively fast, by (2) the low difference thresholds and, thus, by (3) the mean Weber fraction being lower than Weber fractions in timing experiments involving other animals, and by (4) the Weber fraction being lower than Weber fractions determined in other sensory experiments in pinnipeds. Future experiments will help to characterise the pinnipeds’ sense of time in more detail and will reveal if seals can extract temporal information not only from visual stimuli but also from stimuli of other modalities. These studies might also pinpoint the role the sense of time plays in the daily lives of pinnipeds.

## Electronic supplementary material

Below is the link to the electronic supplementary material.

Supplementary file1 (DOCX 24 kb)
