# Supplementary material for: Visual timing abilities of a harbour seal (Phoca vitulina) and a South African fur seal (Arctocephalus pusillus pusillus) for sub- and supra-second time intervals
Source: Anim Cogn. 2020 May 9;23(5):851–9. doi: 10.1007/s10071-020-01390-3 (PMC7415748; doi:10.1007/s10071-020-01390-3)
Supplement: Supplementary file 1 — Supplementary file1 (DOCX 24 kb) [file 10071_2020_1390_MOESM1_ESM.docx]

Supplementary material for manuscript

VISUAL TIMING ABILITIES OF A HARBOUR SEAL (*PHOCA VITULINA*) AND A SOUTH AFRICAN FUR SEAL (*ARCTOCEPHALUS PUSILLUS PUSILLUS*) FOR SUB- AND SUPRA-SECOND TIME INTERVALS

Tamara Heinrich, Andrea Ravignani, Frederike D. Hanke*

* Author of correspondence:

Frederike D. Hanke, University of Rostock, Institute for Biosciences, Neuroethology, Albert- Einstein-Str. 3, 18059 Rostock, Germany, frederike.hanke@uni-rostock.de

**Supplement Tab.1** Overview of the performance of the **South African fur seal** for all tested standard time intervals (STI, in s) with the sequence of comparison time intervals in **open chamber** **conditions** (CTI, in s). Difference thresholds (∆S, in s) were determined and the Weber fractions calculated as ∆S/STI in the timing experiment. The sessions within which the seal reached the learning criterion for a specific STI/CTI combination are highlighted in bold. The STIs are listed according to the sequence of testing

| STI (s) | CTI (s) | Performance as correct choices (%) | | | | | | | Δs (s) | | | Weber fraction |  |
| --- | --- | --- | --- | --- | --- | --- | --- | --- | --- | --- | --- | --- | --- |
|  |  | 1.session | 2.session | 3.session | 4.session | 5.session | 6.session | |  |  |  |  |  |
| 5 | 15;11^a^ | First pretraining phase during which the animal showed no learning | | | | | | | |  |  |  |  |
|  |  |  |  |  |  |  |  | |  | | |  |  |
| 3 | 11^b^ | Second pretraining phase of 207 trials | | | | | |  | | |  | | |
|  | 11 | **93.3** | **100** | **100^c^** |  |  |  | | 0.32 | | | 0.10 |  |
|  | 9 | **76.67** | **86.67** |  |  |  |  | |  | | |  |  |
|  | 8 | **100** | **86.67** |  |  |  |  | |  | | |  |  |
|  | 7 | **96.67** | **100** |  |  |  |  | |  | | |  |  |
|  | 6 | 70 | **83.34** | **96.67** |  |  |  | |  | | |  |  |
|  | 5 | **83.34** | **83.34** |  |  |  |  | |  | | |  |  |
|  | 4 | **76.7** | **90** |  |  |  |  | |  | | |  |  |
|  | 3.5 | 73.3 | 63.34 | 50 | 70 | **80** | **86.67** | |  | | |  |  |
|  | 3.25 | 66.67 | 70 | 83.34 | 60 | 80 | 70 | |  | | |  |  |
|  |  |  |  |  |  |  |  | |  | | |  |  |
| 5 | 15 | **100** | **93.34** |  |  |  |  | | 0.84 | | | 0.17 |  |
|  | 11 | **93.34** | **93.34** |  |  |  |  | |  | | |  |  |
|  | 8 | **83.34** | **86.67** |  |  |  |  | |  | | |  |  |
|  | 7 | **76.67** | **86.67** |  |  |  |  | |  | | |  |  |
|  | 6 | 63.34 | 70 | **90** | **76.67** |  |  | |  | | |  |  |
|  | 5.5 | 53.34 | 56.67 | 66.67 | 56.67 | 53.34 |  | |  | | |  |  |
|  |  |  |  |  |  |  |  | |  | | |  |  |
| 7 | 11 | **86.67** | **96.67** |  |  |  |  | | 0.77 | | | 0.11 |  |
|  | 9 | 70 | **96.67** | **96.67** |  |  |  | |  | | |  |  |
|  | 8 | 86.67 | 66.67 | **86.67** | **83.34** |  |  | |  | | |  |  |
|  | 7.5 | 63.34 | 66.67 | 46.67 | 70 | 70 |  | |  | | |  |  |
|  |  |  |  |  |  |  |  | |  | | |  |  |
| 12 | 17 | **83.3** | **86.7** |  |  |  |  | | 1.30 | | | 0.11 |  |
|  | 15 | **83.3** | **93.3** |  |  |  |  | |  | | |  |  |
|  | 14 | **90** | **86.7** |  |  |  |  | |  | | |  |  |
|  | 13 | 70 | 70 | 60 | 90 | 56.7 |  | |  | | |  |  |

^a^ We started to train the fur seal with a STI of 5s and a CTI of 15s, the latter being changed to 11s after some time. However, for both STI/CTI combinations, the fur seal did not learn the task. Thus we shifted to a stimulus combination of STI 3s/CTI 11s (see second pretraining phase).

^b^ After the first pretraining phase in which the animal did not learn to discriminate a STI of 5s from a CTI of 15s or 11s, we changed to the STI of 3s and a CTI of 11s. This stimulus combination was learnt by the animal in 207 trials and the difference threshold was determined.

^c^ After the animal had learnt to discriminate a STI of 3s versus a CTI of 11s, one session of overtraining with the fur seal showing a performance of 100% correct choices was conducted for the consolidation of the performance.

**Supplement Tab.2** Overview of the performance of the **South African fur seal** for all tested standard time intervals (STI, in s) with the sequence of comparison time intervals (CTI, in s) in **closed chamber conditions**. Difference thresholds (∆S, in s) were determined and the Weber fractions calculated as ∆S/STI in the timing experiment. The sessions within which the seal reached the learning criterion for a specific STI/CTI combination are highlighted in bold. The STIs are listed according to the sequence of testing.

| STI (s) | CTI (s) | Performance as correct choices (%) | | | | | | Δs (s) | Weber fraction |
| --- | --- | --- | --- | --- | --- | --- | --- | --- | --- |
|  |  | 1.session | 2.session | 3.session | 4.session | 5.session | 6.session |  |  |
| 3 | 5 | **96.67** | **96.67** |  |  |  |  | 0.21 | 0.07 |
|  | 4 | **96.67** | **90** |  |  |  |  |  |  |
|  | 3.5 | 73.34 | 70 | 80 | 73.34 | **90** | **93.34** |  |  |
|  | 3.25 | **80** | **80** |  |  |  |  |  |  |
|  | 3.15 | 73.34 | 60 | 60 | 63.34 | 80 | 66.67 |  |  |
|  |  |  |  |  |  |  |  |  |  |
| 5 | 8 | **90** | **83.34** |  |  |  |  | 0.71 | 0.14 |
|  | 6 | **76.67** | **90** |  |  |  |  |  |  |
|  | 5.5 | 66.67 | 70 | 70 | 76.67 | 63.34 |  |  |  |
|  |  |  |  |  |  |  |  |  |  |
| 7 | 9 | 70 | 73.34 | **90** | **93.34** |  |  | 0.78 | 0.11 |
|  | 8 | **93.34** | **86.67** |  |  |  |  |  |  |
|  | 7.5 | 50 | 60 | 56.67 | 60 | 56.67 |  |  |  |
|  |  |  |  |  |  |  |  |  |  |
| 1.6 | 3 | **76.7** | **93.3** |  |  |  |  | 0.25 | 0.16 |
|  | 2.6 | **96.7** | **100** |  |  |  |  |  |  |
|  | 2.2 | **96.7** | **90** |  |  |  |  |  |  |
|  | 2.0 | **90** | **90** |  |  |  |  |  |  |
|  | 1.8 | 60 | 63.3 | 80 | 73.3 | 73.3 |  |  |  |
|  |  |  |  |  |  |  |  |  |  |
| 0.8 | 1.3 | **96.7** | **80** |  |  |  |  | 0.08 | 0.10 |
|  | 1.1 | 63.3 | 83.3 | 60 | **86.7** | **80** |  |  |  |
|  | 1.0 | **80** | **86.7** |  |  |  |  |  |  |
|  | 0.95 | 60 | 66.67 | 83.34 | 73.34 | **83.34** | **76.67** |  |  |
|  | 0.90 | **76.7** | **86.7** |  |  |  |  |  |  |
|  | 0.85 | 63.3 | 63.3 | 46.7 | 66.7 | 60 |  |  |  |
|  |  |  |  |  |  |  |  |  |  |
| 0.4 | 1.0 | **96.7** | **76.7** | **96.7^a^** | **96.7** |  |  | 0.10 | 0.24 |
|  | 0.6 | **76.7** | **80** |  |  |  |  |  |  |
|  | 0.55 | 73.3 | 50 | 66.7 | 60 | **76.7** | **76.7** |  |  |
|  | 0.5 | 80 | 56.67 | 70 | **76.67** | **80** |  |  |  |
|  | 0.46 | 53.34 | 60 | 43.34 | 56.67 | 50 |  |  |  |
|  |  |  |  |  |  |  |  |  |  |
| 0.2 | 0.4 | **86.7** | **90** |  |  |  |  | 0.10 | 0.48 |
|  | 0.33 | **93.3** | **86.7** |  |  |  |  |  |  |
|  | 0.27 | 50 | 66.7 | 63.3 | 63.3 | 70 |  |  |  |

^a^ Overtraining with a constant performance above 90% correct choices were conducted for the consolidation of the performance, because of a time break between the STI of 0.8s and STI of 0.4s

**Supplement Tab.3** Overview of the performance of the **harbour seal** for the five tested standard time intervals in **closed chamber conditions** (STI, in s) with the sequence of comparison time intervals (CTI, in s). Difference thresholds (∆S, in s) were determined and the Weber fractions calculated as ∆S/STI in the timing experiment. The sessions within which the seal reached the learning criterion for a specific STI/CTI combination are highlighted in bold. The STIs are listed according to the sequence of testing

| STI (s) | CTI (s) | Performance as correct choices (%) | | | | | | Δs (s) | Weber fraction |
| --- | --- | --- | --- | --- | --- | --- | --- | --- | --- |
|  |  | 1.session | 2.session | 3.session | 4.session | 5.session | 6.session |  |  |
| 1.6 | 3 | 59.25^a^ | 70 | 76.7 | **83.3** | **96.7** |  | 0.16 | 0.10 |
|  | 2.6 | **93.3** | **96.7** |  |  |  |  |  |  |
|  | 2.2 | **90** | **93.3** |  |  |  |  |  |  |
|  | 2 | 96.7 | 73.3 | **80** | **93.3** |  |  |  |  |
|  | 1.8 | **83.3** | **83.3** |  |  |  |  |  |  |
|  | 1.75 | 53.3 | 76.7 | 73.3 | 73.3 | 83.3 | 70 |  |  |
|  |  |  |  |  |  |  |  |  |  |
| 0.8 | 1.3 | **86.7** | **96.7** |  |  |  |  | 0.07 | 0.10 |
|  | 1.1 | **86.7** | **86.7** |  |  |  |  |  |  |
|  | 1.0 | **86.7** | **80** |  |  |  |  |  |  |
|  | 0.95 | **80** | **83.3** |  |  |  |  |  |  |
|  | 0.90 | 66.7 | 60 | 70 | **80** | **83.3** |  |  |  |
|  | 0.85 | 63.3 | 66.7 | 70 | 80 | 53.3 |  |  |  |
|  |  |  |  |  |  |  |  |  |  |
|  |  |  |  |  |  |  |  |  |  |
| 0.4 | 0.6 | **83.3** | **86.7** |  |  |  |  | 0.09 | 0.24 |
|  | 0.55 | 73.3 | **83.3** | **93.3** |  |  |  |  |  |
|  | 0.5 | 73.3 | 70 | 80 | 66.7 | **76.7** | **76.7** |  |  |
|  | 0.466 | 66.7 | 66.7 | 66.7 | 56.7 | 66.7 |  |  |  |
|  |  |  |  |  |  |  |  |  |  |
| 0.2 | 0.333 | 80 | 73.3 | **76.7** | **76.7** |  |  | 0.12 | 0.62 |
|  | 0.266 | 50 | 63.3 | 63.3 | 73.3 | 70 |  |  |  |
|  |  |  |  |  |  |  |  |  |  |
| 0.1 | 0.3 | 60 | **83.3** | **83.3** |  |  |  | 0.16 | 1.58 |
|  | 0.25 | 80 | 73.3 | 73.3 | 80 | 60 |  |  |  |

^a^ only 27 trials were conducted, because of the low motivation of the animal after a break of the experiment
